# Visible-Light Active and Magnetically Recyclable Nanocomposites for the Degradation of Organic Dye

**DOI:** 10.3390/ma7054034

**Published:** 2014-05-21

**Authors:** Helin Niu, Qinmin Wang, Hongxia Liang, Min Chen, Changjie Mao, Jiming Song, Shengyi Zhang, Yuanhao Gao, Changle Chen

**Affiliations:** 1 Anhui Province Key Laboratory of Environment-friendly Polymer Materials, School of Chemistry and Chemical Engineering, Anhui University, Hefei 230601, Anhui, China; E-Mails: qinminwang12@gmail.com (Q.W.); hongxia828@gmail.com (H.L.); maocj2011@gmail.com (C.M.); songjm@ustc.edu.cn (J.S.); syzhangi@126.com (S.Z.); 2 CAS Key Laboratory of Soft Matter Chemistry, Department of Polymer Science and Engineering, University of Science and Technology of China, Hefei 230026, Anhui, China; E-Mail: misschen@mail.ustc.edu.cn; 3 School of Chemistry and Chemical Engineering, Xuchang University, Xuchang 461000, Henan, China; E-Mail: yuanhaogao2@gmail.com

**Keywords:** magnetic photocatalysts, synthesis, TiO_2_, visible-light irradiation

## Abstract

Recyclable visible-light photocatalyst Fe_3_O_4_@TiO_2_ with core-shell structure was prepared by a simple synthetic strategy using solvothermal crystallization of titanium precursor on preformed Fe_3_O_4_ nanopartiles. The photo-degradation reaction of neutral red aqueous solution was tested to evaluate the visible-light photocatalytic activity of the as prepared Fe_3_O_4_@TiO_2_ nanoparticles, which show excellent photocatalytic activity compared with commercial P25 catalyst. Moreover, the Fe_3_O_4_@TiO_2_ nanocomposites can be easily separated from the reaction mixture, and maintain favorable photocatalytic activity after five cycles. The high visible light absorption of the Fe_3_O_4_@TiO_2_ nanocomposites may originate from the absence of electronic heterojunction, excellently dispersity and the high specific surface area of the as-synthesized Fe_3_O_4_@TiO_2_ samples.

## 1. Introduction

Pollutions of commercial dyes have become one of the most serious environmental issues. Recently, photo-catalysis using sunlight has emerged as one of the most attractive techniques for various environmental applications such as wastewater treatment. As the most commonly used photo-catalyst, TiO_2_ has been extensively studied for the detoxification of wastewater [1,2]. However, its large-scale application has been limited due to the following disadvantages: (1) The large band gap (*E*_g_ = 3.2 eV) means it can only be excited by ultraviolet (UV) light, which is only about 4% of solar spectrum [3]; (2) The low barrier for the recombination of photogenerated electron-hole pairs reduces the quantum efficiency and the photo-catalytic activity [4]. Extensive efforts have been made to develop TiO_2_ photo-catalysts that can more efficiently utilize solar or indoor light. Some reports have demonstrated that the introduction of nonmetal [5–8], lanthanide ions [9,10], transitional metal ions [11–13], noble metals [14] and metallic oxides [15] into TiO_2_ lattice can reduce the recombination of electron-hole pairs and enhance its absorption in visible region. However, there have been very few examples about the photocatalyst possessing both visible-light activity and magnetic properties.

Core-shell structured materials combine the properties of two or several materials by synergistic effect, which may lead to unique properties. Fe_3_O_4_@TiO_2_ composite system has attracted a lot of attention due to its magnetic property. However, most of the previously reported Fe_3_O_4_@TiO_2_ systems suffer from dramatically reduced photocatalytic activities due to the electron-hole recombination resulting from the electronic heterojunction between the core-shell Fe_3_O_4_@TiO_2_ structures [16,17]. An alternative strategy was developed to solve this issue by coating the Fe_3_O_4_ core with SiO_2_ insulation layer to avoid unfavorable heterojunction and photodissolution. However, the high temperature (around 500 °C) treatment required during the synthesis usually led to the loss of magnetism, change of iron oxide phase or the generation of mixed iron/titanium oxide [18–23]. Moreover, most of these composite systems only respond to UV light irradiation [24–26]. The development of both magnetically and photocatalytically active catalyst systems is very challenging.

**Scheme 1.**
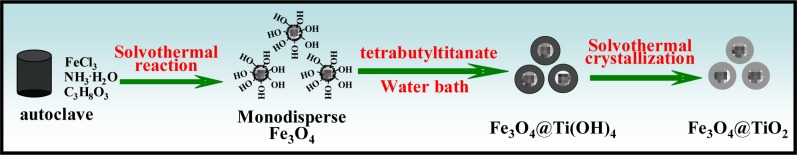
Synthetic route to Fe_3_O_4_@TiO_2_ core-shell nanocomposites.

Herein, we report a novel synthetic route for the preparation of recyclable Visible-light photocatalyst Fe_3_O_4_@TiO_2_ under relatively low temperature (Scheme 1). The method leads to excellently dispersed and homogeneous nanoparticles by avoiding the growing and reuniting of powders. Due to the low synthetic temperature, the magnetic properties and photocatalytic activity of the composites do not deteriorate [27]. The as-prepared materials have higher visible-light absorption and much higher photo-catalytic activity in neutral red decomposition reaction comparing with commercial P25 and can be easily separated and recycled by simple utilization of a magnetic bar.

## 2. Results and Discussion

**Figure 1.**
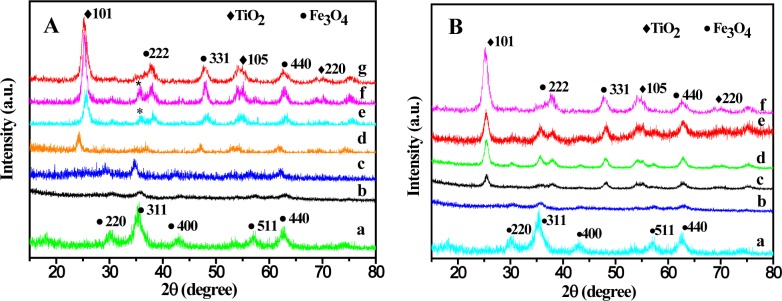
(**A**) XRD spectra of different samples of (a) Fe_3_O_4_ and Fe_3_O_4_@TiO_2_ of prepared at different temperatures of (b) 0 °C, (c) 100 °C, (d) 125 °C, (e) 150 °C, (f) 175 °C, (g) 200 °C; (**B**) XRD spectra of different samples of (a) Fe_3_O_4_ and Fe_3_O_4_@TiO_2_ of prepared at different time under 200 °C of (b) 0 h, (c) 4 h, (d) 6 h, (e) 8 h, (f) 10 h.

**Figure 2.**
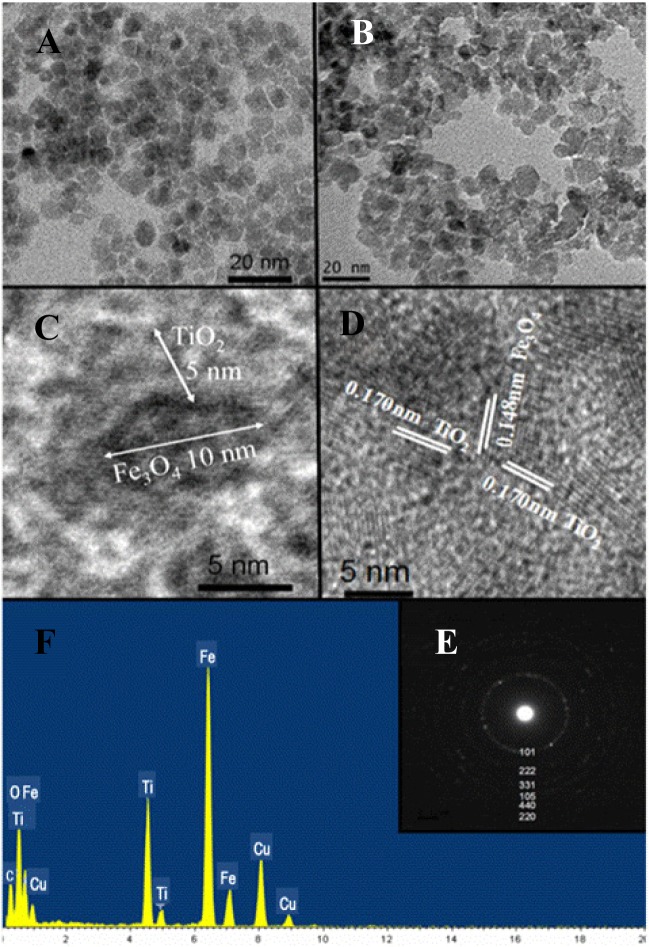
TEM images of (A) the synthesized Fe_3_O_4_ nanocomposites; (B,C) Fe_3_O_4_@TiO_2_ core-shell nanocomposites synthesized by solvothermal crystallization method; (D–F) HRTEM, SAED and EDX spectrum of Fe_3_O_4_@TiO_2_ core-shell nanocomposites.

The XRD patterns of the Fe_3_O_4_ and Fe_3_O_4_@TiO_2_ nanocomposites prepared under different temperature and different time were shown in Figures 1A,B, which showed that the as prepared samples are of high purity. The samples were scanned from 20° to 80°, 2θ degrees using a Cu Kα radiation with a characteristic wavelength (λ) of 0.15405 nm. The Fe_3_O_4_ nanoparticles exhibit a broad peak at about 2θ = 35.2° (Figure 1A,B), which is the (311) reflection and has highly crystalline cublic spinel structure, agreeing well with the standard Fe_3_O_4_ XRD spectrum (JCPDS card No. 89-3854). Figures 1A–b and 2B-b are the XRD patterns of predecessor of Fe_3_O_4_@TiO_2_. The weak diffraction peaks of Fe_3_O_4_ were observed. The diffraction peaks of TiO_2_ are stronger and broader with the increasing of the reaction temperature from Figure 1A(c–g) and the reaction time from Figure 1B(c–f). These peaks can be attributed to (101), (105) and (220) reflections of anatase TiO_2_, agreeing well with the standard anatase TiO_2_ XRD spectrum (JCPDS card No. 89-4203). The iron compound peaks in the XRD patterns became weaker and weaker, due to the formation of the TiO_2_ shell on the surface of iron oxide. Scherrer method was used to determine the grain size Scherrer based on the (101), (331) and (440) reflection equation as follows [28]:

D=Kλ/βcosθ (1)

where *D* is the average diameter of the calculated particles; *K* is the shape factor of the average grain size (the expected shape factor is 0.89); λ is the wavelength characteristic in Å (in this particular case λ =1.5405 Å); β is the width of the X-ray peak at half its high. The average crystallites size of Fe_3_O_4_@SiO_2_ prepared at different temperatures of 200 °C, 175 °C, 150 °C and 135 °C are 18.6 nm, 22.4 nm and 22.8 nm and 24.8 nm. The average crystallites size of Fe_3_O_4_@SiO_2_ prepared at different times at 10 h, 8 h, 6 h, 4 h are 18.6 nm, 19.1 nm, 22.1 nm and 22.6 nm. High temperature and long reaction time lead to smaller crystallites. Based on the XRD spectrum analysis, the optimal solvothermal crystallization condition is 200 °C and 8 h.

TEM image of the Fe_3_O_4_ nanoparticles shows excellent monodispersity with an average diameter of 10 nm (Figure 2A). TEM images of the Fe_3_O_4_@TiO_2_ nanocomposites indicate an average diameter of 20 nm and a typical core-shell structure (Figure 2B,C). The thickness for the black core (Fe_3_O_4_) and shell (TiO_2_) is *ca.* 10 nm and 5 nm. HRTEM analysis shows highly crystalline structure with lattice space of 0.148 nm and 0.17 nm, corresponding to the (440) plane for Fe_3_O_4_ and (105) plane for TiO_2_ (Figure 2D). SAED pattern (Figure 2E) shows a twinned structure with six diffraction rings originating from the TiO_2_ (101), (105), (220) planes and the Fe_3_O_4_ (222), (331), (440) planes, which agrees very well with the XRD patterns. The EDX analysis (Figure 2F) reveals the existence of Fe, Ti, O, C and Cu elements with atomic percentage of 23.46, 10.25, 33.37, 26.13, 6.79, in which C and Cu were from a copper grid with carbon film. The iron peak is weak due to the shielding of the TiO_2_ shell.

**Figure 3.**
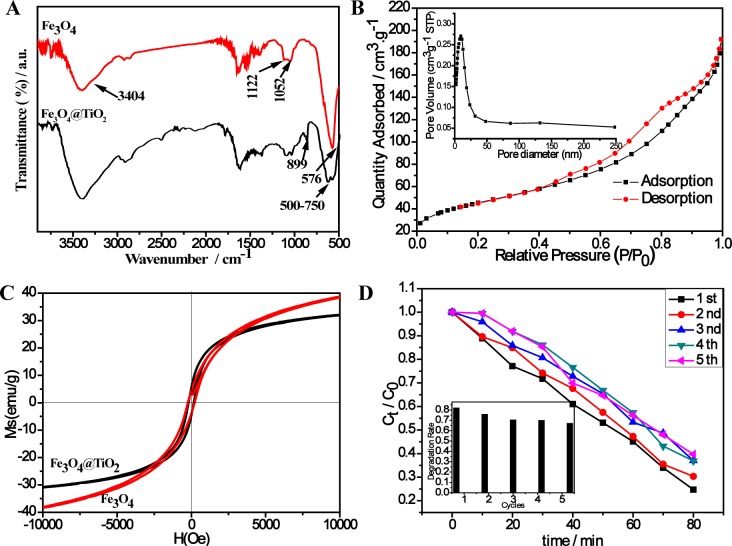
(**A**) FT-IR spectra of the as-made Fe_3_O_4_ nanoparticles (a) and Fe_3_O_4_@SiO_2_ nanoparticles (b); (**B**) N_2_ adsorption and desorption isotherms and pore-size distribution (inset) of the Fe_3_O_4_@TiO_2_; (**C**) M-H curves at room temperature of Fe_3_O_4_@TiO_2_ and Fe_3_O_4_. The insert is the magnetic separation photographs of Fe_3_O_4_@TiO_2_; (**D**) UV-Vis absorption spectrum of the P25 and Fe_3_O_4_@TiO_2_ and the Kulbeka-Munk plot of the energy band gap.

The FT-IR spectrum of the Fe_3_O_4_ nanoparticles (Figure 3A) shows a characteristic band for Fe–O stretching vibration at 576 cm^−1^ [29], the broad bands at *ca.* 3400 cm^−1^ and 1100 cm^−1^ are associated with the O–H stretching and bending vibration [30,31]. These results suggested that the Fe_3_O_4_ surfaces are linked with hydroxyl groups, which not only enables better dispersity of the Fe_3_O_4_ nanoparticles, but also enhances the affinity between the Fe_3_O_4_ nanoparticles and the predecessor TiO_2_. The FT-IR spectrum of the Fe_3_O_4_@SiO_2_ nanoparticles (Figure 3A) shows characteristic absorption peaks at 500–750 cm^−1^ of titania [29], further confirming the successful preparation of the Fe_3_O_4_@TiO_2_ microspheres. Nitrogen adsorption and desorption isotherms were used to investigate the specific surface area and porosity of the as-prepared Fe_3_O_4_@TiO_2_ nanomaterials, and the corresponding N_2_ adsorption-desorption isotherms and pore size distributions are shown in Figure 3B. It can be seen that the samples have type IV isotherms (according to IUPAC classification) [27]. The Brunauer–Emmett–Teller (BET) specific surface area of the as-synthesized Fe_3_O_4_@TiO_2_ samples is 160.474 m^2^·g^−1^ by calculation from nitrogen adsorption. The single point adsorption total pore volume of pores is less than 501.4597 nm diameter. The high specific surface area of the sample nanostructures may provide more active sites for the catalytic reaction.

The magnetic behavior of Fe_3_O_4_@TiO_2_ and Fe_3_O_4_ are investigated using M-H curves from VSM analysis at room temperature (Figure 3C). The saturation magnetization value (*M*_s_), coercivity (*H*_c_), remanent magnetization (*M*_r_) of Fe_3_O_4_@TiO_2_ nanocomposites are 30.75 emu/g, 167.89 Oe and 6.14 emu/g, respectively. In contrast, the *M*_s_, *H*_c_ and *M*_r_ values of the Fe_3_O_4_ are 38.55 emu/g, 229.34 Oe and 4.6 emu/g, respectively. This may due to the lower content of Fe_3_O_4_ in Fe_3_O_4_@TiO_2_. The Fe_3_O_4_@TiO_2_ sample is still considered to be superparamagnetic due to its small *H*_c_ and *M*_r_ value though individual particles may be larger than the superparamagnetic critical size (20 nm) [32]. From the insert in Figure 3C, it can be see that as prepared Fe_3_O_4_@TiO_2_ nanocomposites can be evenly dispersed in water, and easily separated from water upon placing a small magnetic bar at one side of the bottle.

Fe_3_O_4_@TiO_2_ has a broad absorption (200~800 nm) in UV-Vis analysis (Figure 3D). In contrast, commercial P25 shows minimum absorption in the >400 nm range. The energy band gap of the samples as a semiconductor was calculated by the Kulbeka-Munk theory [33]. The relationship between absorption coefficient (α) and the incident photon energy (*h*ν) can be written as α = B_d_ (*h*ν − *E*_g_)^1/2^/(*h*ν), where B_d_ is the absorption constant. The Kulbeka–Munk plot of (α*h*ν)^1/2^ *versus* (*h*ν) was presented in the insert. The extrapolated value of the absorption edge was about 2.10 eV. This value is much narrower than pure TiO_2_ (3.2 eV). The significant absorption in the >400 nm range and the small band gap suggested its possible photocatalytic properties under visible light.

**Figure 4.**
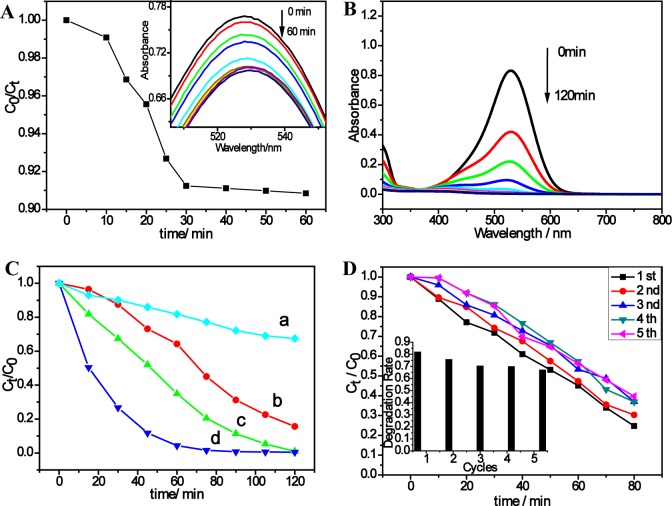
(**A**) The adsorption rate curves of neutral red test in the dark with the presence of Fe_3_O_4_@TiO_2_. The insert is the adsorption curves of neutral red in the dark with the presence of Fe_3_O_4_@TiO_2_ nanocomposites; (**B**) the photodegradation curves of neutral red under visible light in the presence of Fe_3_O_4_@TiO_2_ nanocomposites; (**C**) the photodegradation rate curves of neutral red test in presence of only visible light without catalyst (a) and under the visible light irradiation with Sample-2 (b), P25 (c) and Fe_3_O_4_@TiO_2_ nanocomposites (d); (**D**) the degradation rate of the neutral red by Fe_3_O_4_@TiO_2_ nanocomposites. The insert is the normalized rate constant in different cycles

The photocatalytic properties of Fe_3_O_4_@TiO_2_ composite were investigated with the degradation reaction of neutral red in aqueous solution under 400–700 nm wavelength to mimic sunlight. Figure 4B shows the UV-Vis absorbance peak of neutral red (at 530 nm) [34] gradually decreased with irradiation time and completely disappeared after the reaction. Figure 4A shows that the absorbance of the neutral red solution decreases with the time in the dark. After 30 min, there is no obvious change in absorbance, indicating that equilibrium is reached. Figure 4C shows the concentration profiles of neutral red during the experiment. The degradation is very slow in the presence of only dye and light (without catalyst, Figure 4C(a)). The activity of Fe_3_O_4_@TiO_2_ (Figure 4C(d)) is much higher than that of the commercial catalyst P25 (Figure 4C–d). This is not surprising since Fe_3_O_4_@TiO_2_ demonstrates significant absorption in the visible light region while P25 does not (Figure 3D).

The surprising visible light absorption and the excellent visible light photocatalytic activity might originate from the absence of electronic heterojunction, excellently dispersity and the high specific surface area of the Fe_3_O_4_@TiO_2_ structures. In contrast, Sample-2 was prepared under the same conditions except 10 mL distilled water was used instead of 10 mL urea solution. Sample-2 has much lower activity than P25 or Fe_3_O_4_@TiO_2_ composites, suggesting the crucial role of the urea. The presence of urea during the synthesis may which may introduce N-doping in the Fe_3_O_4_@TiO_2_ composites. It has been shown before that N-doping in TiO_2_ can induce visible light adsorption [35–37].

Recyclability experiment was also studied (Figure 4D). After each experiment, the photocatalysts were separated by applying an external magnetic field, washed three times with ethanol, dried at 60 °C and redistributed in fresh neutral red solution. The catalyst showed favorable reusability after five times of recycling. We did observe some extent of the loss in the catalytic activity after each cycle. The decrease in the degradation rate may be due to the weakening of the absorbance ability of the catalysts or the loss of some catalysts during the collection.

## 3. Experimental Section

### 3.1. Materials

Anhydrous FeCl_3_, ammonia, glycerol, tetrabutyl titanate (TBOT), polyvinylpyrrolidone (PVP), urea, absolute ethanol (C_2_H_5_OH). All chemicals are of analytical grade and used without any purification; Deionized water was used throughout the experiments.

### 3.2. Synthesis

Scheme 1 shows the synthetic route to Fe_3_O_4_@TiO_2_ core-shell nanocomposites. 0.16 g anhydrous FeCl_3_ was fully dispersed in 5 mL ammonia. Fifteen mL glycerol was added to the above mixture, which was transferred into a Teflon-sealed autoclave. The autoclave was heated at 180 °C for 10 h before being cooled in air naturally. The magnetic Fe_3_O_4_ nanoparticles were separated by a magnet and washed three times by distilled water and redispersed in 10 mL absolute ethanol for subsequent processing. Under mechanical stirring, tetrabutyl titanate (TBOT) was diluted in absolute ethanol containing 0.08 g PVP and added to the Fe_3_O_4_ suspension. The mixture was sufficiently mixed and heated in water bath at 80 °C for 15 h. During this process, a mixture of 20 mL ethanol and 10 mL urea were added dropwise. Then, the suspension was aged for 12 h so that Ti(OH)_4_ can adsorb on the surface of the Fe_3_O_4_ nanoparticles. The composite particles were aggregated by an external magnetic field, and washed three times with distilled water and absolute ethanol. The magnetic particles were resuspended in a proper amount of absolute ethanol and the solution was transferred into a Teflon-sealed autoclave. The autoclave was heated to 200 °C for 8 h, and dark brown magnetic nanocomposites were obtained. The final Fe_3_O_4_@TiO_2_ core-shell nanocomposites were separated by magnet, washed three times by ethanol and oven-dried at 60 °C for 4 h. Another Fe_3_O_4_@TiO_2_ core-shell nanocomposites were synthesized by the same method except 10 mL distilled water was used instead of 10 mL urea solution and designated as Sample-2.

### 3.3. Evaluation Photocatalytic Activity

The photocatalytic activity was evaluated by the degradation of neutral red aqueous solution at room temperature. Twenty mg catalysts were suspended in 70 mL dye solution (10 mg·L^−1^). The solution was continuously stirred in the dark for 30 min. Then, the solution was exposed to visible light from a 30 W xenon lamp (PLS-SXE 300, Beijing, China, and the UV light was filtered by the filter). The water samples were collected by magnetic field every 15 min to measure the concentration of dye solution with UV-Vis spectra. After the reaction, the magnetic catalysts were collected using a permanent magnetic field, washed with ethanol for 3 times, dried in oven at 60 °C for 4 h, and rested for the recyclability experiment. The concentration change (*C*/*C*_0_) and the percent conversion ((*C*−*C*_0_)/*C*_0_) of the dye were calculated and plotted *vs.* irradiation time.

### 3.4. Characterization

X-ray powder diffraction (XRD) of Fe_3_O_4_ and Fe_3_O_4_@TiO_2_ core-shell nanocomposites were obtained using X-ray diffractometry (XRD, Rigaku D/max-RA, graphite monochromatized CuKα radiation, λ = 1.5406 Å, at 36 kV and 25 mA). Transmission electron microscopy (TEM), High resolution transmission electron microscopy (HRTEM) and energy-dispersive X-ray (EDX) analysis were obtained by a JEM-2100 transmission electron microscope (accelerating voltage = 200 kV). IR spectra were measured by using KBr pellets on a NECUS-870. The ultraviolet-visible (UV-Vis) diffuse reflectance spectroscopy and the UV-Vis absorption of the products were recorded on a UV spectrometer (UV-1750). Magnetic properties of samples were evaluated on a BHV-55 vibrating sample magnetometer (VSM).

## 4. Conclusions

In summary, Recyclable visible-light active photocatalyst Fe_3_O_4_@TiO_2_ with core-shell structure was prepared by a simple synthetic method under mild conditions. The as-prepared nanocomposites were characterized by XRD, TEM, UV-Vis and magnetic analysis, which showed great dispersity, high crystallinity and good magnetic property. Very surprisingly, the nanocomposites demonstrate significant absorption in the Visible light region and is much more active than commercial P25 in the neutral red decomposition reaction. More importantly, the photocatalyst can be easily separated by an external magnetic field and reused. After five cycles, the catalyst still maintained great catalytic activities. This method provides a simple while general strategy to improve the photocatalytic properties of TiO_2_ under Visible light, and introduces magnetic properties at the same time.
